# Circulation of Coxsackievirus A6 in hand-foot-mouth disease in Guangzhou, 2010-2012

**DOI:** 10.1186/1743-422X-11-157

**Published:** 2014-09-01

**Authors:** Biao Di, Ying Zhang, Huaping Xie, Xiaoquan Li, Chun Chen, Peng Ding, Peng He, Dahu Wang, Jinmei Geng, Lei Luo, Zhijun Bai, Zhicong Yang, Ming Wang

**Affiliations:** Guangzhou Center for Disease Control and Prevention, NO.1, Qide Road, Baiyun District, Guangzhou, Guangdong 510440 China

**Keywords:** Hand, Foot and mouth disease, Coxsackievirus A6, Epidemiologic feature, Molecular analysis

## Abstract

**Background:**

Hand, foot and mouth disease (HFMD) is usually caused by Enterovirus 71(EV71), and Coxsackievirus A16 (CV-A16) in Guangzhou, the biggest city of South China. However, Coxsackievirus A6 (CV-A6) were observed increased dramatically from 2010–2012.

**Methods:**

In order to understand and to describe the epidemiologic and genetic characteristics of CV-A6, specimens of 5482 suspected HFMD cases were collected and examined by real-time fluorescence PCR. All samples positive for enteroviruses were analyzed by descriptive statistics. Phylogenetic analysis of CV-A6 based on the VP1 sequences was performed to investigate molecular and evolutionary characteristics.

**Results:**

Coxsackievirus A6 increased dramatically from 9.04% in 2010 to 23.21% in 2012 and became one of the main causative agents of HFMD in Guangzhou. CV-A6 attack rates were highest in one to two year olds (33.14%). Typical clinic symptoms of CV-A6 HFMD include fever (589/720, 81.81%), maculopopular rash and vesicular exanthema around the perioral area (408/720, 56.66%), intraoral (545/720, 75.69%), the buttock (395/720, 54.86%), the trunk (244/720, 33.89%), the knee (188/720, 26.11%), and the dorsal aspects of hands (437/720, 60.69%). Phylogenetic analysis showed the CV-A6 isolates in this study belonged to Cluster A1 and were similar to those found in Shanghai in 2011 and 2012 (JX495148, KC414735), Shenzhen in 2011 (JX473394), Japan in 2011 (AB649243, AB649246), France in 2010(HE572928), Thailand in 2012(JX556564) and Israel in 2012 and 2013(.KF991010, KF991012).

**Electronic supplementary material:**

The online version of this article (doi:10.1186/1743-422X-11-157) contains supplementary material, which is available to authorized users.

## Background

Hand, foot and mouth disease (HFMD) is a common epidemic causing childhood infection caused by enteroviruses. The causative enteroviruses include Coxsackievirus A (2–8, 10, 12, 14, 16), Coxsackievirus B (2, 5) and enterovirus (EV) 71. EV 71 and CV-A16 are the most common. HFMD primarily afflicts infants and children younger than 5 years. Primary symptoms are fever, rash or herpes of the hand, feet and oral cavity. HFMD was first described in 1957 by Seddon in New Zealand. In 1958, CV-A16 was isolated by Robinson et al. in Canada [1]. In 1959, the disease was formally named HFMD. EV71 was isolated in 1969 and determined to be a HFMD causing agent in 1972 [2]. Since then, numerous EV71 and CV-A16 caused HFMD outbreaks have been reported [3].

In China the first case of HFMD was reported in Shanghai in 1982, followed by reports in more than ten provinces and cities, including Beijing, Hebei, Tianjin, Shandong and Guangdong. These reports included several outbreaks of epidemic proportions [4–6]. The etiology of HFMD in China is similar to that described in many other countries of Asia and the world. The major HFMD causing pathogen was CV-A16 in the 1980s and EV71 in the 1990s but caseloads remained low until 2008 when a nationwide epidemic occurred. Subsequently HFMD was named a C-class infectious disease by the national government [7, 8]. In Guangzhou, HFMD associated morbidity is higher than the national average [9]. In addition to high rates of EV71 and CV-A16 infections, CV-A6 has also emerged as a major disease causing strain based on our surveillance. In order to fully understand the epidemic and to develop appropriate control strategies, this paper analyzed the molecular epidemiology and disease characteristics of HFMD caused by CV-A6 in Guangzhou over the period from 2010 to 2012.

## Results and discussion

From January 2010 to December 2012, a total of 5482 suspected HFMD cases were identified, 4111 (75%) of which were positive by the pan-EV test (pan EV fluorescent kit from Guangzhou Huayin Medical Technology Inc.). Of the 4111 pan-EV positive cases, the most frequently presented serotypes were EV71 (1443, 35.10%), CV-A16 (1261, 30.67%), and CV-A6 (720, 17.51%) and untyped enteroviruses (604, 14.69%). EV71 and CV-A16 co-infection cases were 83 (2.02 %).

### Distribution of CV-A6 by year

**Figure 1:**
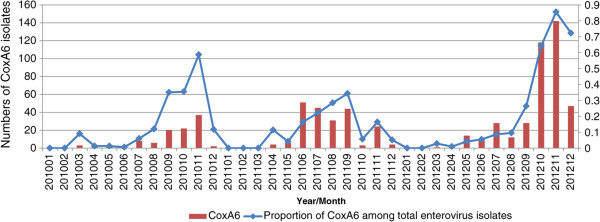
**Monthly distribution of 720 CV-A6 isolates from 2010 to 2012 in Guangzhou, China.**

**Figure 2:**
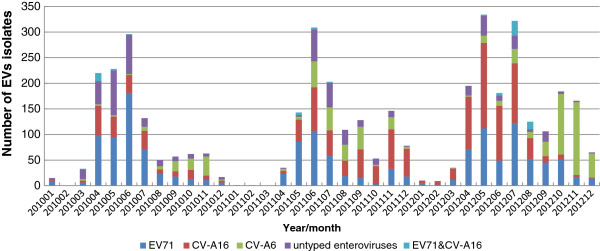
**Distribution of EVS types in hand-foot-mouth diseases patients during 2010 to 2012, Guangzhou.**

HFMD cases were reported in every month except for February, 2010 and January and March, 2011. The proportion of CV-A6 infections increased each year from 9.04% (106/1173) in 2010 to 17.58% (212/1206) in 2011 and 23.21% (402/1732) in 2012. CV-A6 was the main cause of autumn peaks in 2010 and 2012 respectively, while distributed evenly from July to September and reappeared in November, 2011. The proportion of CV-A6 among the total enterovirus types attained peak in November, 2012. Compared to EV71 and CV-A16, the prevalent season for CV-A6 was warm season in 2010 and 2011, while was cool season in 2012 (Figure 1).The predominant autumn pathogen switched from CV-A6 in 2010 to CV-A16 in 2011, and switched to CV-A6 again in 2012. The proportion of HFMD cases positive for enterovirus varied significantly between years, as did the proportion of each serotype. The enterovirus positive rate was 94.73% (1173/1243) in 2010, 58.85% (1206/2049) in 2011 and 79.01% in 2012 (1732/2192). EV71 and CV-A16 remained the most frequent serotypes in each year. Conversely the proportion of other EVs decreased each year from 24.72% (290/1173) in 2010 to 15.26% (184/1206) in 2011 and to 7.51% (130/1732) in 2012. EV71 and CV-A16 co-infection increased from 0.91% to 3.06% (51/1732) in 2012 (Figure 2).

### Distribution of CV-A6 infection and disease characteristics

Of the total 4111 pan-EV positive HFMD cases, 3547(86.28%) patients were recorded with age and gender, ranging from 1 month to 30 years (median: 2 years) and 1827(52.94%, 1827/3451) were male.

**Figure 3:**
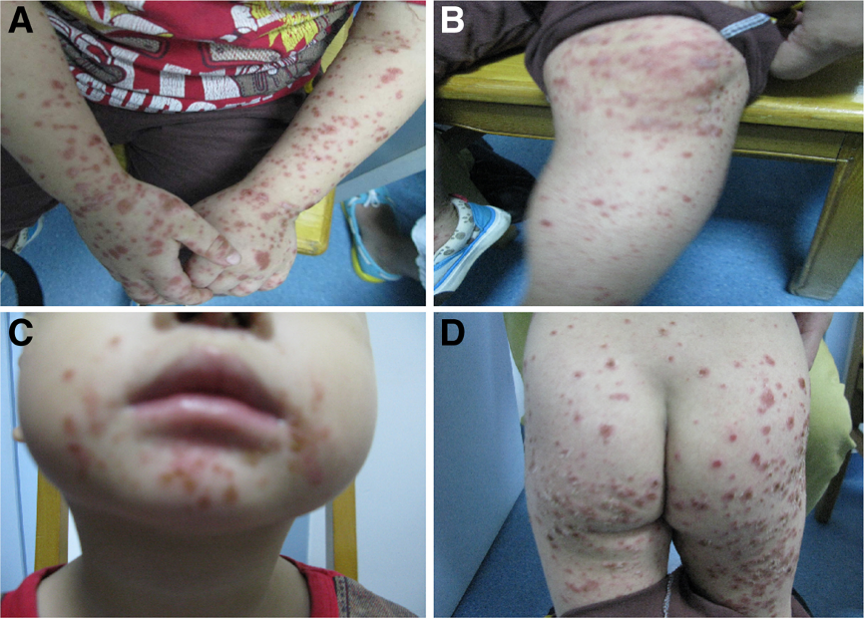
**A 2-year-8-month-old boy experienced prominent skin eruption and vesicles with CV-A6 infection in Guangzhou, 2010–2012. (A)** Eruptions on his hand; **(B)** Eruptions on his foot(especially on his knees); **(C)** Eruptions around his mouth; **(D)** Eruption and vesicles on his buttocks.

Among the patients positive for EVs, 77.54% were aged between one and four. Significant differences in attack rates for each EV serotype were observed for each age group. CV-A6 (173/522, 33.14%) was most common in children aged one to two, however EV71 (376/1281, 29.35%) and CV-A16 (376/1086, 34.62%) were most common in children aged three to four (*χ*^*2*^ = 118.818,*P*<0.05). The ratio of males to females positive for CV-A6 was 1.72:1 and no significant difference for each EV serotype (*χ*^*2*^ = 0.708,*P>*0.05, Table 1).Typical Clinical signs and symptoms of HFMD caused by CV-A6 were fever (560/720, 77.78%), vesicular eruptions around the perioral area (408/720, 56.66%), intraoral (545/720, 75.69%), the buttock (395/720, 54.86%), the trunk (244/720, 33.89%), the knee (188/720, 26.11%), and the dorsal aspects of hands (437/720, 60.69%) in addition to the usual manifestation of skin eruptions on the hands, feet, and mouth. (Figure 3) According to our follow up survey by phone, some recovered patients had nail loss 1 month after initial symptoms. Most cases of HFMD were self-limited and only 8 of 31 severe cases were CV-A6 infection which suffered from meningitis. None CV-A6 infected patient died during 3 years. Other 23 severe cases were EV71 positive.

**Table 1:** **The demographic of patients with different enteroviruses infection from 2010 to 2012**

Virus			EV71				CV-A16				CV-A6				EV71and CV-A16 co-infection				Untyped EV			
		V-P	P	Cons ratio (%)	M	F	P	Cons ratio (%)	M	F	P	Cons ratio (%)	M	F	P	Cons ratio (%)	M	F	P	Cons ratio (%)	M	F
Age year	0-	219	69	5.39	43	26	46	4.24	33	13	39	7.47	28	11	7	8.54	5	2	58	10.07	34	24
	1-	718	264	20.61	176	88	150	13.81	95	55	173	33.14	103	70	17	20.73	9	8	114	19.79	75	39
	2-	714	270	21.08	173	97	208	19.15	123	85	115	22.03	70	45	19	23.17	13	6	102	17.71	59	43
	3-	1063	376	29.35	245	131	376	34.62	229	147	117	22.41	74	43	27	32.93	15	12	167	28.99	101	66
	4-	504	177	13.82	113	64	192	17.68	127	65	48	9.20	33	15	9	10.98	7	2	78	13.54	41	37
	5-	163	64	5.00	37	27	61	5.62	40	21	13	2.49	11	2	1	1.22	0	1	24	4.17	15	9
	≥6	166	61	4.76	33	28	53	4.88	30	23	17	3.26	11	6	2	2.44	0	2	33	5.73	23	10
Total		3547	1281	36.12	820	461	1086	30.62	677	409	522	14.72	330	192	82	2.31	49	33	576	16.24	348	228
Ratio (M/F)			1.78				1.66				1.72				1.48				1.53

### Phylogenetic analysis

The 344bp VP1 region (2561nt-2904nt, responding to AY421764) were sequenced from CV-A6 isolates and 72 representative isolates were selected to perform phylogenetic analysis. Among those isolates, 43 were selected to represent 22 clustered cases and outbreaks in different locations and times, as well as 29 were selected from sporadic cases, including 8 severe infections (Additional file 1: Table S2). The sequences were submitted to GenBank under accession numbers (KF535162-KF535186, KF639916-KF639926, KM262854-KM262889). The sequence of 38 additional isolates, representing the full CV-A6 geographic and time diversity and previously reported isolates from China and the world, were downloaded from GenBank and were subjected to phylogenetic analysis together with our isolates.

**Figure 4:**
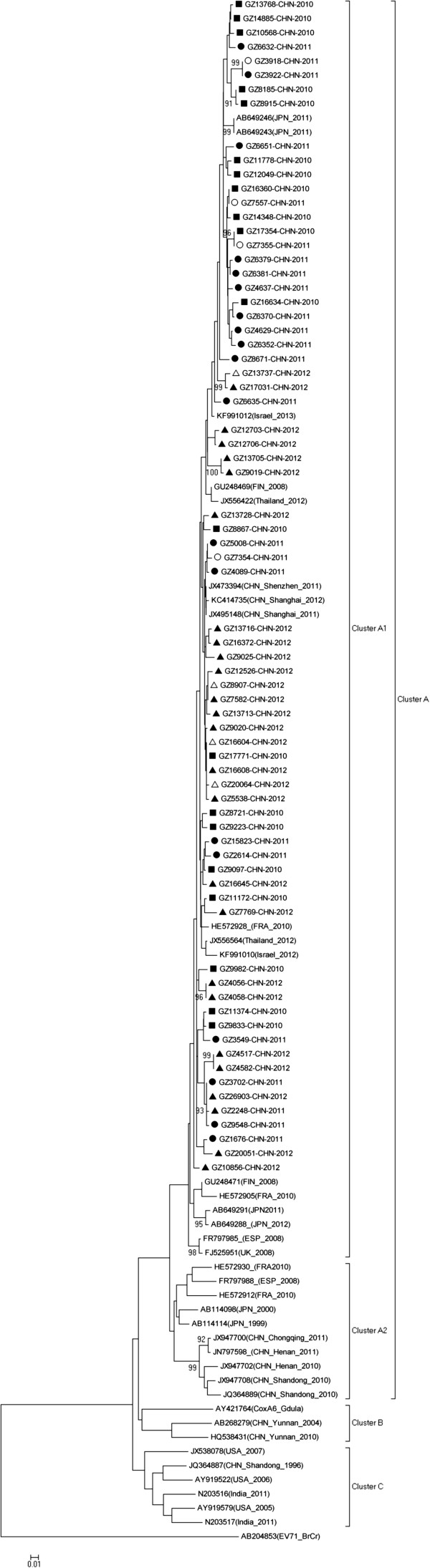
**Phylogenetic analysis of 72 Guangzhou CV-A6 isolates from 2010–2012 (■ 2010 isolates ● 2011 isolates ▲ 2012 isolates ○2011 severe cases ∆ 2012 severe cases) and 38 reference isolates from other Chinese and international locations based on partial VP1 nucleotide sequences.** The dendrogram was generated by the neighbor-joining method (bootstrap analysis with 1,000 pseudo replicate data sets) by Mega 5.0. Genotypes are shown to the right of the tress and bootstrap values are shown at each major node. The scale at the bottom indicates a measurement of relative phylogenetic distance.

Across all three years the nucleic acid and deduced amino acid homology of 72 CV-A6 subtypes was 89.25%~100% and 92.92%~100%, respectively. The nucleic acid sequences of 44 isolates from 2010 and 2011 had higher homology (92.31~100% identity), whereas the nucleotide identities between 2012 and 2010, 2012 and 2011 isolates were 89.91%~99.4% and 89.25%~100%, respectively. Meanwhile, the deduced amino acid identities of 2010 and 2011 (93.51%~100%) were also higher than that of 2011 and 2012 (92.92%~100%). All CV-A6 isolates of this study belonged to cluster A1, similar to those found in Shanghai 2011 and 2012, Shenzhen 2011, Thailand 2012, Israel 2012 and 2013, Japan 2011 and France 2010. Our isolates were not similar to those of Chongqing 2011, Henan 2010–2011 and Shandong 2010, which were found by colleagues in Shandong province from 2009–2011 [10], neither to the isolates in Yunnan 2004 nor Yunnan 2010 which fell into cluster B in our analysis (Figure 4).

This epidemiological investigation and molecular phylogenetic analysis indicate that the HFMD epidemic of Guangzhou, is becoming more severe and dynamic in terms of the number and types of causative EVs. In 2011, CV-A6 cases appeared primarily in the summer (June-September). However, the CV-A6 outbreak-periods were significantly different in both 2010 and 2012 and were focused in autumn and winter, with a large peak in the winter of 2012. Since 2008, CV-A6 has caused outbreaks in Spain, France, Finland, Israel, UK, Japan, Singapore, USA and Taiwan [11–16]. From 2009, numerous non-linked reports of infections caused by CV-A2, CV-A4, CV-A5, CV-A6, CV-A9, CV-A10 CV-B3 and CV-B5 have been released in Shandong, Henan, Chongqing, Changchun and Qingdao [17–22]. However, to our knowledge, this is the first time that CV-A6 was found emerged dramatically as a predominant causative pathogen of HFMD in a metropolis of China. And this may suggest that other EVs, like CV-A6, could become dominant in the future through introduction to the local population and adaption to the local environment.

Additionally, CV-A6 attack rates were highest in one to two year olds (33.14%), younger than EV71 and CV-A16 cases which had peak attack-rates in three to four year-olds (29.35% and 34.62%, respectively). This CV-A6-associated younger cohort poses a risk because younger children have low-resistance and auto-immunity and may present with more complex symptoms. This stresses the importance of maintained surveillance of HFMD infection and education on the signs and symptoms of HFMD in younger children.

We found no phylogenetic explanation for the increase in the number of CV-A6 cases in 2011 and 2012. However we hypothesize that the lack of immunity to this serotype in Guangzhou population contributed to the increased caseload. Additionally, Guangzhou has a sub-tropical climate meaning that autumn and winter are relatively mild. This likely allows the persistent transmission of some EV serotypes, such as CV-A6 throughout the year.

The 8 VP1 sequences separated by the severity of symptoms infected with CV-A6 virus were also included in the Cluster A1 with high homology (89.25%~100%) and were mixed throughout the phylogenetic tree. This indicated that differences in the VP1 region cannot account for differences in severity of disease. Although all CV-A6 isolates of this study were classified into cluster A1, the alignment of 2010–2011 isolates showed a higher nucleic acid and amino acid sequence homology than that of 2012. This could suggest a slight evolutionary shift from 2010–2011 to 2012.

A retrospective analysis of the HFMD associated symptoms showed that although there were some severe cases, a majority of patients had a typical presentation with symptoms including fever, upper respiratory tract symptoms, maculopapular rash and vesicular exanthema covering the hands, feet and mouth. Although no deaths associated with CV-A6 infections was found, there were still 8 (8/720, 1.11%) patients who were identified with CV-A6 positive, non-bacterial meningitis. This suggests that CV-A6 not only causes common HFMD but can also severely affect the central nervous system. Different from HFMD caused by EV71 or CV-A16, the patients infected by CV-A6 had intraoral lesions and maculopapular on knees obviously in our study.

The timing and presentation of CV-A6 outbreaks have varied in different geographical regions, suggesting some adaptability of the virus. In the Boston HFMD outbreak caused by CV-A6 in the winter of 2012, perioral papules and perirectal eruption exhibited as the characteristic feature in patients [13]. But perirectal eruption was not observed obviously in Finland series CVA6 outbreaks in 2008 and Taiwan CVA6 outbreak in 2010[13, 23]. In our study, perirectal eruption was not found visibly.

Similar to Taiwan surveillance from 2004–2009, during which only 3 (2.4%) of the 141 inpatients with CV-6 infection had central nervous system involvement [24], only 8 (8/720, 1.11%) of the 720 CV-A6 infectors had meningitis and all recovered uneventfully in our present study. However, an unusually severe outbreak in Nevada, US occurred from November, 2011 to February, 2012 and led to a 19% hospitalization rate [14]. Cases from the 2010 outbreaks in Spain and Finland presented with onychomadesis [11, 23, 25], and outbreaks in France (2010) and Japan (2012) CV-A6 cases were also associated with herpangina and onychomadesis [12, 13]. But unless specified, onychomadesis were often missed because it occurred 1–2 months after infection. These diversities of HFMD presentation demonstrate the importance of symptom monitoring and the physicians should be aware of the emerging pathogens.

An EV71 vaccine is now in stage 3 clinical trials and has proven safe and efficacious for EV71 prevention. However, there is no evidence that it will provide immunity to other EVs such as CV-A16 and CV-A6 [26]. Our research shows that there are many other non-EV71 HFMD-causing EVs that should not be disregarded. Infections caused by more recently detected serotypes such as CV-A6 are not only on the rise but can be associated with severe symptoms. Therefore, even if the EV71 vaccine is highly effective, the number and severity of HFMD cases may not decrease significantly. It is absolutely necessary that future vaccines should target CV-A6, the potential major causative pathogen of HFMD. The molecular epidemiology of CV-A6 should also be enhanced as it is highly likely that this virus will continue to contribute significantly to the HFMD case load. Genetic information from various geographical regions will help determine links to severity and pathogenecity as well as evidence for genetic recombination events which could lead to further outbreaks. Improved surveillance of this emerging virus is therefore warranted.

## Methods

### Sample collection

Feces, stool, throat swabs and cerebrospinal fluid samples were collected from HFMD cases presenting at Guangzhou from 5 surveillance hospitals (Guangdong Women and Children Hospital, Guangzhou Yuexiu District Children Hospital, Zengcheng People’s Hospital, Guangzhou Huado Distrit People’s Hospital, and Huangpu Branch of the First Affiliated Hospital, Sun Yat-sen University) and 12 districts CDCs in Guangzhou. Samples were stored at -20°C and tested at the district level center for disease control and prevention in Guangzhou. From March 1^st^, 2010 to October 30^th^, 2012, 5482 samples were collected. Among the specimens, 5 cerebrospinal fluid specimens were collected from fatal cases, 31 specimens were available from severe cases, including throat swabs and stool, 5444 samples were from mild outpatients, including feces, stool and throat swabs. All patients (or the guardians of the children patients) gave oral informed consent.

### Pretreatment of the samples and extraction of nucleic acid

Feces (1.5-2g) and stool samples were dissolved in 2ml physiological saline (75% NaCl) and vortexed for 10 min, followed by centrifugation at 3,800 rcf/min for 5 min. The supernatant was collected and used for RNA extraction. Throat and anal swabs were vortexed in 2ml physiological saline, centrifuged and the supernatant was used to extract RNA. RNA was extracted from 140 μL of pretreated sample using the QIAamp Viral RNA Mini Kit (Qiagen, Hilden, Germany) and eluted in 50 μL elution buffer. Throat swab and cerebrospinal fluid specimens were not pre-treated prior to extraction.

### CV-A6 detection and genotyping

The collected samples were amplified using the pan EV fluorescent kit (Guangzhou Huayin medical technology Inc.) for enterovirus detection. A positive sample was defined as any sample with a cycle threshold (Ct) value ≤35. Positive samples were further sub-typed using an EV71/CV-A16 RNA fluorescent PCR combo-test kit and a CV-A6 fluorescent PCR kit (Guangzhou Huayin medical technology Inc.). All tests were performed with the 7500 Fast Real-Time PCR system (Applied Biosystems, USA).

For genotyping CV-A6 isolates, the same RNA extracts were used and RT-snPCR was performed on the partial 5’ region of the VP1 capsid protein, as described by Nix [27] with minor modifications. The first cycle of amplification was performed using the SuperScript® One-step RT-PCR Kit (Invitrogen, USA) and 10 pmol of each reverse transcription primer (AN32, AN33, AN34 and AN35) and PCR primers (222, 224), 12.5μl 2xPCR reaction buffer, 1μl SuperScript® RT/Platinum Taq buffer, 2.5 μl template and ddH_2_O to 25μl. Reaction conditions were as follows: incubation at 50°C for 30 min; denaturation at 94°C for 2 min; 30 cycles of denaturation at 94°C for 30 s, annealing at 42°C for 30 s, and extension at 60°C for 45 s; 10 cycles of denaturation at 94°C for 30 s, annealing at 50°C for 30 s, and extension at 68°C for 45 s; final extension at 72°C for 10 min.

The second cycle of amplification was performed with the PrimeScript One-Step RT-PCR kit (Takara, Japan), 1 μl of the RT-PCR product, 0.8 μl of the primer pair AN88, AN89 (20 μM), 25 μl of a 2xPCR buffer, and ddH_2_0 up to 50 μl. Reaction conditions were as follows: 40 cycles of denaturation at 98°C for 10 s, annealing at 60°C for 20 s, and extension at 72°C for 35 s; final extension at 72°C for 7 min. After purification and collection, the correctly sized PCR products were purified with QIAquick PCR Purification Kit (Qiagen, Hilden, Germany). The amplicons were sequenced with primers AN233 and AN232 by using an ABI 3130 Genetic Analyzer (Applied Biosystems, USA). Sequences were confirmed using BLAST in NCBI.

### Statistical analysis

Descriptive statistics were performed, and a database was developed for the storage and analysis of study data. The mobidity and constituent ratio were calculated and analyzed by SPSS 13.0 and Microsoft Excel 2007. The statistical differences between proportions were tested by chi-squared test or non-parametric test. A P-value <0.05 was regarded as statistically significant.

### Analysis of pathogen evolution

Representative isolates were selected for VP1 nucleic acid sequencing, following the method described above. Sequence data for each isolate was formatted and compiled into contiguous segments using Editseq and Seqman programs (DNASTAR, Mdison, WI). Each virus was analyzed for molecular evolution using Cluster W ((Mega5.0) and a phylogenetic tree was produced using the neighbor joining (Mega5.0) method as described by Oberste et al. Seventy-two CV-A6 VP1 sequences from the present study and 38 VP1 sequences from CV-A6 isolates available in the GenBank database were used and displayed as a dendrogram containing 110 isolates. The robustness of the analysis was confirmed by bootstrap analysis with 1,000 pseudo replicates. The sequences of EV71 strain BrCr (U22521) was used as outgroup in the phylogenetic analysis.

Ethics statement: Data was collected as part of government mandated health surveillance and analyzed anonymously so ethical approval was not needed.

## Author’s information

Dr. Biao Di is a researcher who works in the Virology and Immunology laboratory of the Center for Disease Control and Prevention. His research interests focus on the molecular epidemiology of human enterovirus.

## Electronic supplementary material

Additional file 1: Table S2: Information of 72 representative CV-A6 isolates. (DOCX 36 KB)
