# Supplementary material for: Circulation of Coxsackievirus A6 in hand-foot-mouth disease in Guangzhou, 2010-2012
Source: Virol J. 2014 Sep 1;11:157. doi: 10.1186/1743-422X-11-157 (PMC4169826; doi:10.1186/1743-422X-11-157)
Supplement: Supplementary file 1 — Additional file 1: Table S2: Information of 72 representative CV-A6 isolates. (DOCX 36 KB) [file 12985_2014_2491_MOESM1_ESM.docx]

Additional file 1: Table S2 Information of 72 representative CV-A6 isolates

| NO. | Strain | Accession number | Collection date | Source of case | Numbers of Patients identified by lab | Case location |
| --- | --- | --- | --- | --- | --- | --- |
| 1 | GZ17771-CHN-2010 | KM262862 | Dec-10 | Sporadic | 1 | urban |
| 2 | GZ17354-CHN-2010 | KF535176 | Dec-10 | Sporadic | 1 | urban |
| 3 | GZ16634-CHN-2010 | KF535177 | Nov-10 | Outbreak | 10 | nursery school |
| 4 | GZ16360-CHN-2010 | KF535178 | Nov-10 |  |  |  |
| 5 | GZ14885-CNH-2010 | KF535179 | Nov-10 | Sporadic | 1 | urban |
| 6 | GZ14348-CHN-2010 | KF535180 | Oct-10 | Sporadic | 1 | urban |
| 7 | GZ13768-CHN-2010 | KF535181 | Oct-10 | Sporadic | 1 | urban |
| 8 | GZ12049-CHN-2010 | KF535182 | Sep-10 | Sporadic |  | urban |
| 9 | GZ11778-CHN-2010 | KF535183 | Sep-10 | Clustered | 5 | Primary school |
| 10 | GZ11374-CHN-2010 | KM262861 | Sep-10 | Clustered | 4 | Primary school |
| 11 | GZ11172-CHN-2010 | KM262860 | Sep-10 | Sporadic | 1 | urban |
| 12 | GZ10568-CHN-2010 | KF535184 | Aug-10 | Sporadic | 1 | urban |
| 13 | GZ9882-CHN-2010 | KM262859 | Aug-10 | Clustered | 5 | nursery school |
| 14 | GZ9833-CHN-2010 | KM262858 | Aug-10 |  |  |  |
| 15 | GZ9223-CHN-2010 | KM262857 | Jul-10 | Sporadic | 1 | urban |
| 16 | GZ9097-CHN-2010 | KM262856 | Jul-10 | Clustered | 6 | community |
| 17 | GZ8915-CHN-2010 | KF535185 | Jul-10 |  |  |  |
| 18 | GZ8867-CHN-2010 | KM262855 | Jun-10 | Sporadic | 1 | urban |
| 19 | GZ8721-CHN-2010 | KM262854 | May-10 | Sporadic | 1 | nursery school |
| 20 | GZ8185-CHN-2010 | KF535186 | May-10 | Sporadic | 1 | urban |
| 21 | GZ1676-CHN-2011 | KM262863 | Apr-11 | Sporadic | 1 | urban |
| 22 | GZ2248-CHN-2011 | KM262864 | May-11 | Sporadic | 1 | urban |
| 23 | GZ2614-CHN-2011 | KM262865 | May-11 | Sporadic | 1 | urban |
| 24 | GZ3549-CHN-2011 | KM262866 | May-11 | Sporadic | 1 | urban |
| 25 | GZ3702-CHN-2011 | KM262867 | Jun-11 | Sporadic | 1 | urban |
| 26 | GZ3918-CHN-2011^p^ | KF639916 | Jun-11 | Outbreak | 20 | primary school |
| 27 | GZ3922-CHN-2011 | KM262868 | Jun-11 |  |  |  |
| 28 | GZ4089-CHN-2011 | KM262869 | Jun-11 |  |  |  |
| 29 | GZ4629-CHN-2011 | KF639917 | Jun-11 | Outbreak | 10 | nursery school |
| 30 | GZ4637-CHN-2011 | KF639918 | Jun-11 |  |  |  |
| 31 | GZ5008-CHN-2011 | KM262870 | Jul-11 | Sporadic | 1 | urban |
| 32 | GZ6352-CHN-2011 | KF639919 | Jul-11 | Outbreak | 21 | community |
| 33 | GZ6370-CHN-2011 | KM262871 | Jul-11 |  |  |  |
| 34 | GZ6379-CHN-2011 | KF639920 | Jul-11 |  |  |  |
| 35 | GZ6381-CHN-2011 | KF639921 | Aug-11 |  |  |  |
| 36 | GZ6635-CHN-2011 | KM262872 | Aug-11 | Clustered | 8 | primary school |
| 37 | GZ6632-CHN-2011 | KF639922 | Aug-11 |  |  |  |
| 38 | GZ6651-CHN-2011 | KF639923 | Aug-11 | Sporadic | 1 | urban |
| 39 | GZ7354-CHN-2011^p^ | KM262873 | Sep-11 | Clustered | 4 | family |
| 40 | GZ7355-CHN-2011^p^ | KF639924 | Sep-11 |  |  |  |
| 41 | GZ7557-CHN-2011^p^ | KF639925 | Sep-11 | Clustered | 3 | nursery school |
| 42 | GZ8671-CHN-2011 | KF639926 | Nov-11 | Sporadic | 1 | urban |
| 43 | GZ9548-CHN-2011 | KM262875 | Nov-11 | Clustered | 3 | nursery school |
| 44 | GZ15823-CHN-2011 | KM262874 | Nov-11 | Sporadic | 1 | urban |
| 45 | GZ26903-CHN-2012 | KM262889 | Dec-12 | Sporadic | 1 | urban |
| 46 | GZ20064-CHN-2012^p^ | KF535162 | Dec-12 | Clustered | 9 | community |
| 47 | GZ20051-CHN-2012 | KF535163 | Dec-12 |  |  |  |
| 48 | GZ17031-CHN-2012 | KF535164 | Nov-12 | Clustered | 5 | nursery school |
| 49 | GZ16645-CHN-2012 | KM262888 | Nov-12 | Outbreak | 20 | primary school |
| 50 | GZ16608-CHN-2012 | KF535165 | Nov-12 |  |  |  |
| 51 | GZ16604-CHN-2012^p^ | KF535166 | Nov-12 |  |  |  |
| 52 | GZ16372-CHN-2012 | KM262887 | Nov-12 | Sporadic | 1 | urban |
| 53 | GZ13737-CHN-2012^p^ | KF535167 | Oct-12 | Outbreak | 18 | nursery school |
| 54 | GZ13728-CHN-2012 | KF535168 | Oct-12 |  |  |  |
| 55 | GZ13716-CHN-2012 | KF535169 | Oct-12 | Outbreak | 15 | nursery school |
| 56 | GZ13713-CHN-2012 | KF535170 | Oct-12 |  |  |  |
| 57 | GZ13705-CHN-2012 | KF535171 | Sep-12 | Sporadic | 1 | urban |
| 58 | GZ12706-CHN-2012 | KF535172 | Sep-12 | Clustered | 9 | nursery school |
| 59 | GZ12703-CHN-2012 | KF535173 | Sep-12 |  |  |  |
| 60 | GZ12526-CHN-2012 | KM262886 | Aug-12 | Sporadic | 1 | urban |
| 61 | GZ10856-CHN-2012 | KM262885 | Aug-12 | Sporadic | 1 | urban |
| 62 | GZ9025-CHN-2012 | KM262884 | Aug-12 | Outbreak | 18 | primary school |
| 63 | GZ9020-CHN-2012 | KF535174 | Jul-12 |  |  |  |
| 64 | GZ9019-CHN-2012 | KF535175 | Jul-12 |  |  |  |
| 65 | GZ8907-CHN-2012^p^ | KM262883 | Jul-12 | Sporadic | 1 |  |
| 66 | GZ7769-CHN-2012 | KM262882 | Jul-12 | Clustered | 3 | family |
| 67 | GZ7582-CHN-2012 | KM262881 | Jun-12 | Clustered | 2 | family |
| 68 | GZ5538-CHN-2012 | KM262880 | Jun-12 | Sporadic | 1 | urban |
| 69 | GZ4582-CHN-2012 | KM262879 | May-12 | Clustered | 4 | community |
| 70 | GZ4517-CHN-2012 | KM262878 | May-12 |  |  |  |
| 71 | GZ4056-CHN-2012 | KM262876 | Apr-12 | Outbreak | 1 | family |
| 72 | GZ4058-CHN-2012 | KM262877 | Apr-12 |  | 1 |  |
